# Biomonitoring with Micronuclei Test in Buccal Cells of Female Farmers and Children Exposed to Pesticides of Maneadero Agricultural Valley, Baja California, Mexico

**DOI:** 10.1155/2016/7934257

**Published:** 2016-02-14

**Authors:** Idalia Jazmin Castañeda-Yslas, María Evarista Arellano-García, Marco Antonio García-Zarate, Balam Ruíz-Ruíz, María Guadalupe Zavala-Cerna, Olivia Torres-Bugarín

**Affiliations:** ^1^Facultad de Ciencias, Universidad Autónoma de Baja California, 22800 Ensenada, BC, Mexico; ^2^Centro de Investigación Científica y de Educación Superior de Ensenada, 22800 Ensenada, BC, Mexico; ^3^Escuela de Ciencias de la Salud, Universidad Autónoma de Baja California, 22800 Ensenada, BC, Mexico; ^4^School of Medicine, Universidad Autónoma de Guadalajara, 44100 Guadalajara, JAL, Mexico

## Abstract

Feminization of the agricultural labor is common in Mexico; these women and their families are vulnerable to several health risks including genotoxicity. Previous papers have presented contradictory information with respect to indirect exposure to pesticides and DNA damage. We aimed to evaluate the genotoxic effect in buccal mucosa from female farmers and children, working in the agricultural valley of Maneadero, Baja California. Frequencies of micronucleated cells (MNc) and nuclear abnormalities (NA) in 2000 cells were obtained from the buccal mucosa of the study population (*n* = 144), divided in four groups: (1) farmers (*n* = 37), (2) unexposed (*n* = 35), (3) farmers' children (*n* = 34), and (4) unexposed children (*n* = 38). We compared frequencies of MNc and NA and fitted generalized linear models to investigate the interaction between these variables and exposition to pesticides. Differences were found between farmers and unexposed women in MNc (*p* < 0.0001), CC (*p* = 0.3376), and PN (*p* < 0.0001). With respect to exposed children, we found higher significant frequencies in MNc (*p* < 0.0001), LN (*p* < 0.0001), CC (*p* < 0.0001), and PN (*p* < 0.004) when compared to unexposed children. Therefore working as a farmer is a risk for genotoxic damage; more importantly indirectly exposed children were found to have genotoxic damage, which is of concern, since it could aid in future disturbances of their health.

## 1. Introduction

Health risk associated with different labors is related to contact with corrosive, infectious, carcinogen, cytotoxic, mutagenic, or genotoxic agents. Research around genetic toxicology and risk assessment or workplace exposure is important since exposure to several hazardous agents is common and could aid in health issues; agricultural activity is often associated with exposure to high volumes of pesticides, mainly organochlorines, organophosphorus, carbamates, pyrethroids, and various inorganic compounds, which are used to control pests in the agriculture zone of Baja California, Mexico [1]. Such chemical agents are an important source of soil and water contamination that will have an impact on living organisms' health, including humans. Pesticides can enter the body by three routes: oral and respiratory routes and contact with skin and mucosal tissue. Pesticide exposure can cause acute poisoning with nausea, vomiting, headache, chest pain, eye, skin, and throat irritation and additionally can cause potential long term health effect with allergies, cancer, nervous system damage, birth defects, and infertility [2]. Adverse effects of pesticides depend on the dose, route, and type; since some can have metabolites that accumulate and persist in living organisms, additionally toxic effects of these compounds are associated with malnutrition and dehydration [3]. Furthermore some pesticides have been tested individually by* in vitro* genotoxicity testing methods and considered as potential chemical mutagens [4].

Women that work as farmers in the agricultural valley in Maneadero, Baja California, usually are coming from the south of the country, most of them from indigenous ancestors with scarce education, and most of them are not provided with medical insurance or social security, even though they are continuously exposed to pesticides [5, 6]. Hazardous exposition to pesticides has been previously documented in the valley of Salinas located in Baja California [7].

In Maneadero valley children are indirectly exposed to pesticides through contact with their farmer mothers and the environment, since most of them live less than 500 m from the agricultural areas. Children are especially vulnerable to adverse effects from pesticides, from conception through birth, due to their constant growth and excessive proliferation rate. Furthermore, early in life exposure to pesticides may be particularly detrimental given that children do not have adult level of enzymes to detoxify until after age of 7 [8], especially for organophosphate pesticides. Additionally during this age there is a rapid and formative brain development which can result affected after pesticide exposure if there are genetic variants that result in decreased PON1, an enzyme related to detoxification [9].

Several studies have evaluated pesticides effects in offspring from farmers and described the presence of neurological damage, respiratory affection, birth defects, diabetes, cardiovascular diseases, hormonal problems, and genotoxicity [10–13]. To evaluate genotoxic damage, the methodology frequently used is expensive and complicated and usually involves invasion; opposite to this, the micronuclei test (MN test) performed in buccal mucosa cells is a precise, inexpensive, noninvasive, and easy method for measuring DNA damage and cell death in the oral epithelium not requiring cell cultures [14–17]. Furthermore, an increased frequency of MN is an efficient biomarker used for the diagnosis of genomic instability, since MN presence represents fragments or whole chromosomes that failed to join the nucleus during cell division and are considered excellent markers of genotoxicity [15, 18].

Nuclear abnormalities (NA) are additional biomarkers that can be recognized through the performance of the MN test; these abnormalities can occur during cell differentiation, indicating DNA damage, cytotoxicity, or cell death when observed in high frequencies [19–21]. Such NA can be identified in the cytoplasm or in the nuclei itself; they consist of binucleated cells (BN), lobulated nuclei (LN), karyorrhexis (KR), condensed chromatin (CC), pyknotic nuclei (PN), and karyolysis (KL). Molecular mechanisms that drive the presence of each of these NA are not well understood, neither is their biological significance in terms of cell function.

There has been a clear association between pesticide exposure and genotoxic damage previously; nonetheless most studies did not evaluate genotoxicity through the MN test; we believe that since this is a more practical methodology especially for children, our results can be replicated to search for genotoxicity in farmers and their children working in different countries, without the requirement of expensive, more elaborated, and invasive techniques [17–19, 21].

The purpose of the present study was to evaluate the genotoxic effect through the MN test and nuclear abnormalities (NA) in buccal mucosal cells of farmers who work at the agricultural valley of Maneadero and children living in close proximity to the fields.

## 2. Methods

### 2.1. Ethical Considerations

This study was approved by the research and bioethics committee of Universidad Autonoma de Baja California with registry number 5-031-074-07-001. The study complied with Mexican Research Regulations and the Declaration of Helsinki. Each participant gave their informed consent before sampling.

### 2.2. Study Population

Study subjects were selected in an intentional nonprobabilistic way, and only after acceptance via informed consent they were included in the study; cases were all encountered, working in the agricultural fields of Maneadero, Baja California, being constantly exposed to several groups of pesticides. Controls were unexposed women selected from a distant population (16 km) in Ensenada, Baja California, who were healthy housewives or black coated workers. We divided the study population into four groups: (1) farmers, (2) unexposed, (3) farmers' children, and (4) unexposed children. Study subjects were interviewed to obtain personal and clinical information according to predesigned questioner, including the following: age, alimentary habits, smoking, alcohol intake, and time of exposure to pesticides. Subjects with history of cancer, radiotherapy, current infections, and dental procedures within the previous month were excluded from the study.

### 2.3. Cell Collection and Staining

Each participant rinsed their mouth with water to keep it clean and exfoliated buccal mucosal cells were collected through a smear of the inside lining of the subjects' oral mucosa of both cheeks. The smears were directly transferred to slides previously coded and dried at room temperature. Afterwards, these slides were fixed with 80% ethanol for 48 h and stained with acetoorcein for 2 h and fast green for 10 minutes as previously described [15, 19, 21–23].

### 2.4. MN and NA in Exfoliated Buccal Cells

The cells in the oral mucosa samples were analyzed using a Carl Zeiss IVFL Axiostar Plus microscope with the objective 100x/1.25. The MNC score was quantified manually and blindly by the same observer, for a total of 2000 cells in order to identify both normal cells and abnormal MN or other NA, using the HUMNxl scoring criteria [17–19, 21], briefly described in here:* Normal cells* (NC) with the nucleus are uniformly stained, oval- or circle-shaped, and smaller than the cytoplasm. There is absence of any other structure besides the nucleus that contains DNA; these cells are considered as completely differentiated cells.* Micronucleated cells* (MNc) are characterized by the presence of a main nucleus and smaller structures denominated micronuclei (MN). One MN has circle or oval shape and its length is between 1/3 and 1/16 of the main nucleus; the intensity of the stain, texture, and plane is equal in both structures. MN characteristics are as follows: rounded smooth perimeter suggestive of a membrane, less than a third the diameter of the associated nucleus, but large enough to discern shape and color; staining intensity similar to that of the nucleus; similar texture to that of nucleus; same focal plane as nucleus; and absence of overlap with, or bridge to, the nucleus.* Binucleated cells* (BN) are cells with two main nuclei, and usually both nuclei are in close proximity or even in contact, both with similar shape and stain to a normal nucleus. BN formation seems to be related to interference during the procedures at the end of cell division. Karyorrhexis (KR) is characterized by nuclear chromatin aggregation and fragmented cell membrane, visualized as a denominated nuclear spotted pattern, indicative of nuclear fragmentation which will lead to nuclear disintegration. Condensed chromatin (CC) is when the nuclei look intensively stained, with chromatin aggregates and intact cell membrane, a denominated nuclear spotted or nuclear striated pattern. It is evident that chromatin is aggregated in some nucleus regions, while other areas lack the presence of chromatin. When the condensation is extended, it appears like a fragmented nucleus; these just like karyorrhexis cells end up with nuclear fragmentation, which leads to eventual disintegration of the nucleus. Cells with pyknotic nuclei (PN) have a nucleus diameter of approximately 1/3 of normal nucleus, with high density of nuclear material which is uniformly distributed, but highly stained. Karyolysis (KL) is when the nucleus has complete lack of DNA which probably represents an advanced stage of cellular death.* Nuclear Buds* (NBUDs): the nucleus presents a strong constriction in one extreme, suggestive of nuclear elimination material process by budding formation. The lobule has similar characteristics in morphology to the nucleus but it is smaller (1/3 to 1/4 of the main nucleus).

### 2.5. Statistical Analysis

For descriptive purposes about age and exposure years results are expressed as mean (standard deviation); indigenous language, education, daily income, time of exposition, smoking, drinking, and diet habits are expressed as percentages; MNc and NA counts are expressed as mean (standard deviation) and reported as the number of occurrences per 2000 cells. Except for age, the distribution of the variables was not able to pass normality test (Shapiro-Wilks test *p* > 0.01); after this and in order to establish inferences about the risk of farmers to develop MNc and NA we used the Mann-Whitney *U* tests. To analyze factors that can be associated with increased MNc we determined the OR and performed Fisher exact test. A *p* value of <0.05 was considered to be statistically significant. All statistical analyses were conducted with the Statistical Program GraphPad Prism V5.

## 3. Results and Discussion

### 3.1. Demographics and General Characteristics of Study Population

Maneadero valley is located 10 km at the south of Ensenada with parcel extension of 4,200 hectares in which 40% of the obtained products are produced the entire year and 60% are seasonal. Main commercial activity in this region is agriculture and products obtained from this region include tomatoes, potatoes, zucchinis, cilantro, lettuce, onions, grapes, strawberry, olive, asparagus, green tomatillo, barley, alfalfa, rye, and flowers. Different pesticides are used in this zone and are described in Table 1.

In the present study a total of 144 women and their offspring were included; mean age of farmers (*n* = 37) was 35.5 ± 12.4 years, and that of unexposed women (*n* = 35) was 31.9 ± 10.6. For their children, mean age of farmer's children (*n* = 34) was 7.8 ± 3.2 years and that of children from unexposed women (*n* = 38) was 10.3 ± 3.4 years. There was significant difference between ages of exposed and unexposed groups; this is an important consideration since it has been documented that aging is associated with increased frequencies of MN [14]. Farmers' years of exposure were 7.7 ± 8.7, ranging from 1 to 25 years; other characteristics of the examined population are represented in Table 2.

### 3.2. Frequency of MNc and NA in Farmers

**Figure 1:**
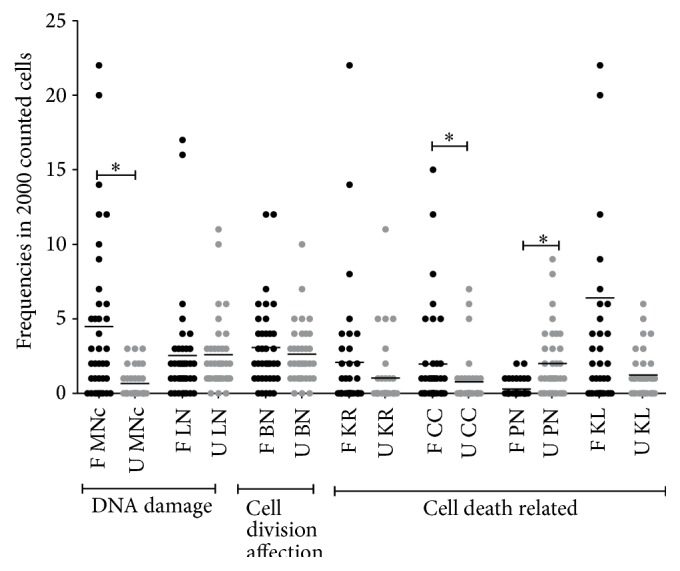
Frequencies of micronucleated cells and nuclear abnormalities in farmers and unexposed women. F = farmers; U = unexposed; MNc = micronucleated cells; LN = cells with lobulated nucleus; BN = binucleated cells; KR = karyorrhexis; CC = cells with condensed chromatin; PN = pyknotic cells; and KL = karyolysis. ^*∗*^ *p* < 0.05.

We compared frequencies of MNc and NA between farmers and unexposed women and found significant differences in MNc (*p* < 0.0001), CC (*p* = 0.0376), and PN (*p* < 0.0001) represented in Table 3; more detailed information about dispersion of values is evident in Figure 1; as noticed the dispersion is bigger in the farmers group when compared to unexposed women, except for the nuclear abnormality PN, which is cell death related. Furthermore values obtained from farmers represent the highest frequencies in MNc and NA, when compared to all other groups, except for PN which was more frequently found in the unexposed group.

The presence of MNc in the exposed group reflects both genotoxic damage and cell death. With respect to NA, some authors suggest that the presence of binucleated cells (BN) is indicative of genotoxic damage, while pyknosis, condensed chromatin, karyorrhexis, and karyolysis are originated after cytotoxic damage [17].

Even though the clinical significance of NA presence has not been fully elucidated, an honest attempt has been made by previous researchers to try to understand their biological meaning; all of these NA have been associated with the presence of diverse chronic and degenerative diseases, as well as with exposition to hazardous chemicals. In general, the presence of BN is a reflection of failure of cytokinesis due to either defects in formation of the microfilament ring or cell cycle arrest due to malsegregation of chromosomes or telomere dysfunction [17]. The presence of MNc and LN is related to chromosomal instability or DNA damage, while CC, KR, PN, and KL are associated with cell death.

Previous studies in Mexico evaluating genotoxic damage after pesticide exposure were mostly performed in lymphocytes, and significant increased frequencies of MNc were found in exposed subjects [1, 24–26]. Only a few studies have evaluated buccal mucosa cells, and results were contradictory [27, 28]. Internationally, Bolognesi et al. performed a literature review to analyze the association between MN presence and pesticide exposure; they found 18 studies performed in lymphocytes of which 9 had positive results and 9 could not sustain an association; additionally 13 studies were performed in buccal mucosa cells, of which only 5 demonstrated an association between MNc and pesticide exposure [4].

During the present study we were interested in a small subgroup of farmers that were found to have >5 MNc (14 out of 37 women); this subgroup could be at risk of developing health related issues. Because of this, we searched for other factors that could be responsible for higher MNc frequencies, such as alcohol consumption and tobacco use, living in close proximity to the fields (<500 mts^2^), time of exposition to pesticides (>5 years), and increased age (>35 years); we found that none of these factors, previously associated with increased MNc frequency, was significantly associated with higher frequencies of MNc; results from this analysis are represented in Table 4.

There are only a few studies that assess the distances where people involved in farming are living; one of these studies was carried out by Lee and colleagues [29] after reviewing agricultural activity in 11 states of the USA; they concluded that farmers as well as people who live in close proximity to the fields (<400 m^2^) are at risk of suffering from the adverse events of pesticides due to contamination of the air, water, and soil. Although a significant association between living in close proximity to the field and high MNc frequencies was not observed in the present study, during sampling we found families living very close to the Maneadero agricultural valley, right outside the fields, which is of concern, since most of these subjects will suffer in the future from several types of affection that have been consistently implicated with pesticides.

When we evaluated frequencies of MNc in association with time of exposure to pesticides, we found that there was a slight increase in MNc frequencies after acute exposition <5 years (5.0 ± 6.7) when compared to subjects with >5 years of exposition to pesticides (3.9 ± 4.1). Even though this difference was nonsignificant, this finding could reflect the natural history of pesticide exposure, with an exacerbated response during the acute exposition, and later, during a chronic low level exposure to pesticides, the induction of an adaptive response related to an increase in apoptosis sensitivity and/or a more extended cell cycle delay enables appropriate DNA repair and therefore a decrease in MNc frequency [30].

### 3.3. Frequency of MNc and NA in Children

**Figure 2:**
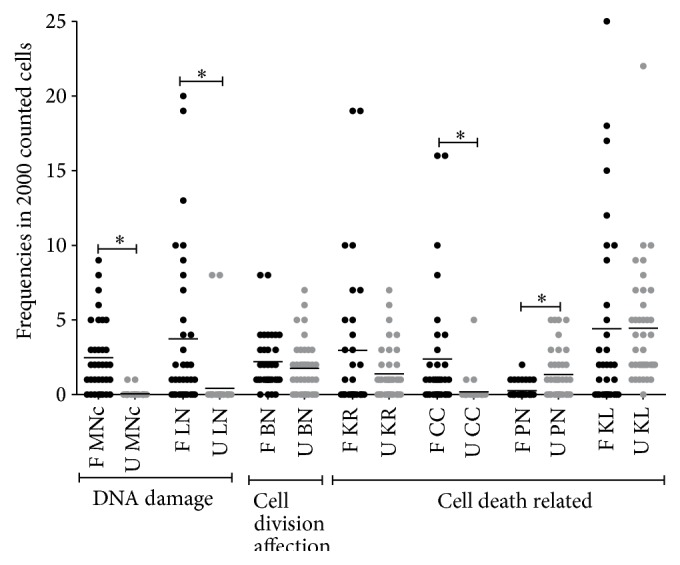
Micronuclei and nuclear abnormalities frequencies in indirectly exposed children and unexposed children. MNc = micronucleated cells; LN = cells with lobulated nucleus; BN = binucleated cells; KR = karyorrhexis; CC = cells with condensed chromatin; PN = pyknotic cells; and KL = karyolysis. ^*∗*^ *p* < 0.05.

With respect to children, the highest frequencies of MNc were found in farmers' children (*p* < 0.0001), when we analyzed NA and increased frequency of some of them was also evident: LN (*p* < 0.0001), CC (*p* < 0.0001), and PN (*p* = 0.0043). These differences can be found in Table 3 and the dispersion of values in Figure 2. In accordance with our findings, other studies in Mexico and South America with children being exposed to pesticides found increased frequency of MNc, even though NA can differ in results [31–33].

The induction of MNc formation after indirect exposure to pesticides might be due to the fact that farmers' homes are located in close proximity to the fields and additionally by contact with clothes or personal objects impregnated with pesticides. Children are especially susceptible to genotoxic damage due to differences in the mutagens metabolism, distribution, and excretion, when compared to adults [34]. Additionally we should consider, as suggested by Holland et al., that in children cell division dynamics are different when compared to adults, since it has been demonstrated that cellular migration occurs in a different rate influenced by the age and hormonal differences [12].

Our findings suggest that in farmers' children there is a chromosomal instability represented by increased frequencies of MNc and NL; additionally they could suffer from cytotoxicity, since CC represents cells that are no longer transcriptionally active and undergoing apoptosis. The presence of PN represents an irreversible condensation of chromatin; this NA is often found with CC and KR although their biological significance is not clear [17].

In order to clearly identify the genotoxic damage that these children are exposed to, future research is needed, where information about the degree of exposition is registered, including lifestyle, nutrition, and immune function in order to be able to elucidate mechanisms for micronuclei generation during childhood and adult life.

## 4. Concluding Remarks

Farmers are at risk of developing genotoxic damage secondary to pesticide exposition; more importantly their children who are living in close proximity to the fields demonstrate this genotoxic damage. Children can be easily monitored for genotoxic damage through the MN test; it would be of extreme importance to develop more studies with prospective design in order to obtain information about relative risks and even more important to design strategies directed towards the prevention of future genotoxic damage of the identified population with higher MNc and NA frequencies, given the fact that they are susceptible to health related issues.

## Figures and Tables

**Table 1:** Main pesticides used in Baja California, Mexico.

Mechanism of action	Uses	Chemical group	Concentration %, EPA classification^a^ (toxicity^b^)
Organophosphate			
Inhibits acetylcholinesterase and is a DNA alkylating agent, classified as carcinogenic, mutagenic, and teratogenic	Insecticide	Diazinon	25–90, not likely (IV)
		Azinphos-methyl	35, not likely (I)
		Malathion	90, evidence (IV)
		Dimethoate	40, C (II)
		Methamidophos	39.6–48.3, E (IB)
	Herbicide	Bensulide	12.5, not likely (III)
		Glyphosate	48, E (IV)

Carbamate			
Rapid onset; inhibits acetylcholinesterase and other enzymes	Insecticide	Methomyl	90, E (IB)
	Insecticide	Oxamyl	24–42, E (IA)
	Fungicide	Mancozeb	56.4–80, B2 (III)
	Fungicide	Maneb	75–80, B2 (III)

Organochlorines			
GABA receptor antagonist inhibits Ca^2+^, Mg^2+^ channels.	Insecticide	Endosulfan	25–48, not likely (I)

Pyrethroid			
Affects Na^+^ channels	Insecticide	Permethrin	34–48, C (IB)
		Bifenthrin	10, C (II)

Biperidiles			
Interferes in electrons transference and inhibits the reduction of NADP to NADPH during photosynthesis, with superoxide radical formation	Herbicide	Paraquat	24, C (IA)

Others			
Mechanism of action not clearly stablished	Fungicide	Copper oxychloride	85.0, D (III)
	Fungicide	Chlorothalonil	54, likely (IV)

^a^Chemicals Evaluated for Carcinogenic Potential, Science Information Management Branch, Health Effects Division, Office of Pesticide Program, US Environmental Protection Agency (2006) [35]. A, human carcinogen; B, probable human carcinogen; B1, limited evidence of carcinogenicity from epidemiological studies; B2, sufficient evidence from animal studies; C, possible human carcinogen; D, not classifiable as to human carcinogenicity; E, evidence of noncarcinogenicity for humans; nd, no data available; evidence, suggestive evidence of carcinogenicity, but not sufficient to assess human carcinogenic potential; likely, likely to be carcinogenic to humans; not likely, not likely to be carcinogenic to humans. ^b^World Health Organization Classification of Pesticides by Hazard: IA—extremely hazardous; IB—highly hazardous; II—moderately hazardous; III—slightly hazardous [36].

**Table 2:** Social and demographic characteristics of farmers from the Maneadero valley and unexposed women.

		Farmers (*n* = 37)	Unexposed (*n* = 34)
		%	%
Indigenous language	Yes	27	24
	Not	73	76

Education	None	58	5
	Basic	23	19
	Middle	4	14
	Higher	15	62

Daily income	MXP	$115.5	$189.5

Time of residence in sampled locations	1 to 5 years	30	36
	6 to 10 years	5	9
	10 or more years	61	55

Years of exposition to pesticides	1 to 5 years	51	NA
	6 to 10 years	22	
	10 to 25 years	27	

Smoke	Yes	6	0
	Not	94	100

Alcohol	Yes	6	9
	Not	94	91

Fruit intake (per week)		3.00 ± 2.27	5.09 ± 1.30

Vegetable intake (per week)		2.63 ± 2.67	5.18 ± 1.94

Additional supplements (vitamins)	Yes	24	0
	Not	15	100
	Unanswered	3	—

MXP: Mexican pesos; NA: nonapplicable.

**Table 3:** Frequencies of micronuclei and nuclear abnormalities in farmers from the Maneadero valley and children.

	Women (*n* = 71)			Children (*n* = 72)		
	Farmers (*n* = 37)	Unexposed (*n* = 34)	*p* value	Farmers (*n* = 34)	Unexposed (*n* = 38)	*p* value
Age	35.5 ± 12.4	27.7 ± 9.4	0.0047	7.8 ± 3.2	10.3 ± 3.4	0.0111
MNc	4.5 ± 5.5	0.7 ± 0.9	<0.0001	2.5 ± 2.5	0.1 ± 0.2	<0.0001
LN	2.5 ± 3.7	2.6 ± 2.5	0.3512	3.7 ± 5.3	0.7 ± 2.3	<0.0001
BN	3.1 ± 2.9	2.6 ± 2.1	0.7317	2.2 ± 2.0	2.3 ± 2.0	0.2833
KR	2.1 ± 4.4	1.0 ± 2.3	0.2480	3.0 ± 5.1	1.0 ± 1.2	0.9863
CC	2.0 ± 3.4	0.8 ± 1.7	0.0376	2.4 ± 4.2	0.1 ± 0.3	<0.0001
PN	0.3 ± 0.6	2.0 ± 2.3	<0.0001	0.3 ± 0.5	1.4 ± 1.8	0.0043
KL	6.4 ± 12.1	1.2 ± 1.6	0.0558	4.4 ± 6.4	4.3 ± 3.1	0.0617

MNc = micronucleated cells; LN = lobulated nucleus; BN = binucleated cells; PN = pyknotic cells; CC = condensed chromatin; KR = karyorrhexis; KL = karyolysis. Results are presented as mean ± SD from number of occurrences per 2000 counted cells. *p* value was obtained with the Mann-Whitney *U* test.

**Table 4:** Risk factors that could increase MNc frequency in farmers (*n* = 37).

Variable	Samples	>5 MNc	<5 MNc	OR (CI 95%)	*p* value
	*n* (%)	*n* (%)	*n* (%)		
Alcohol consumption and tobacco use	5 (14)	3 (8)	2 (5)	5.36 (0.74–38.70)	0.1102
Living in close proximity to fields (<400 m^2^)	21 (57)	7 (19)	14 (38)	2.17 (0.46–10.20)	0.4613
Time of exposition to pesticides (>5 years)	18 (49)	5 (14)	13 (34)	1.07 (0.25–4.59)	1.000
Age (>35 years of age)	11 (30)	5 (14)	6 (16)	3.50 (0.75–16.27)	0.1249

MNc: micronucleated cells, CI: confidence interval. Fisher's exact test (two-sided); a *p* value < 0.05 would have been considered significant.
